# Biology of Incidental Catch Sea Star* Stellaster childreni* Gray, 1840 (Echinodermata: Asteroidea), from Malaysian Borneo Exclusive Economic Zone

**DOI:** 10.1155/2017/1489360

**Published:** 2017-06-11

**Authors:** Ruhana Hassan, Suet Yee Lee, Wan Zabidii Wan Morni

**Affiliations:** Faculty of Resource Science and Technology, Universiti Malaysia Sarawak, 94300 Kota Samarahan, Sarawak, Malaysia

## Abstract

Sea star (class Asteroidea, phylum Echinodermata) is one of the most successful marine organisms inhabiting a wide range of habitats. As one of the key stone species, sea stars are responsible for maintaining much of the local diversity of species within certain communities. Malaysian Exclusive Economic Zone (EEZ) Resource Survey had been carried out from 16th Aug to 6th Nov 2015 and one of the invertebrate by-catch organisms is sea star* Stellaster childreni *Gray, 1840. This study documents morphological characters and diet of the sea star, besides providing brief descriptions of the habitats based on particle size analysis and vessel log data sheet. A total of 217 individuals had been examined throughout this study. Fragments of flora and fauna were found in the gut including Mollusca (gastropod, bivalves, and scaphopods), sponge seagrass, and seaweed as well as benthic Foraminifera.* Stellaster childreni *were found at depth of 45 m to 185 m in the South China Sea off Sarawak Malaysia, with various sea bottom substrata. Approximately 41% of* S. childreni *were found at a mixture of sandy and muddy substratum, followed by mixture of sandy and coral (19.3%), muddy substratum (17.5%), coral substratum (11.5%), and sandy areas (10.6%). The widely distributed sea star on different types of sea beds suggested healthy deep sea ecosystem; thus Malaysia should explore further potential fisheries resources in the EEZ off Sarawak coast.

## 1. Introduction

Class Asteroidea, star fish or sea star, is a marine invertebrate that occurs from intertidal to abyssal deep sea area of approximately 6000 m depth [1], which comprises approximately 1900 extant species classified into 36 families and nearly 370 extant genera. Asteroids serve as “keystone species” due to their predatory nature such as* Pisaster* that interacts with* Mytilus* along the coast of North America [2] which had been documented as determining factors of distribution pattern, abundance, and density of marine organisms [3, 4], thus serving as model organisms in relation to fields of community structure [5] and feeding ecology [6]. Furthermore, in evolutionary developmental biology (Evo-devo), Asteroidea received much attention as debates continue for its complex phylogenetic classifications [1].

For Malaysia, documentation on Asteroidea species inhabiting reef areas is available from South China Sea [7], including Archipelago of Beting Patinggi Ali to Pulau Layang-Layang, South China Sea [8], Pulau Sipadan, and Bodgaya [9]. In addition, check lists of Asteroidea from seagrass area in the southern Peninsular Malaysia are also made available by [10]. Documentation on sea stars in neighbouring Singapore had been reported by [11]. Meanwhile, [12] had produced brief documentation on sea stars composition inhabiting deep sea of Malaysian EEZ off Sarawak coast.

The implementation of the EEZ of Malaysia in 1981 had led to the extension of the fishing ground beyond the traditional fishing area. The total EEZ area of Malaysia is 548,800 km^2^, and Sarawak, one of Malaysian states located in Borneo Island, has around 160,000 km^2^ Malaysian EEZ [13].

Since the 1980s, surveys on fisheries resources in Malaysian EEZ are being carried out by the Department of Fisheries, Malaysia (DoF M) for every 5 or 10 years, depending on budget availability and the current needs of assessing resources to formulate sustainable harvesting. In 2015, DoF M had conducted fisheries resource survey in EEZ off Sarawak coast, using MV SEAFDEC 2 (owned by South East Asia Development Centre based at SEAFDEC Training Department, Bangkok, Thailand). The main objective of the 2015 survey was to determine the current status of marine fisheries available in the EEZ, whereas the specific objective of this study is to examine by-catch invertebrate, sea star* S. childreni* morphological and anatomical characters, its diet, and descriptions on the habitats based on vessel's log data sheet.

## 2. Materials and Methods

**Figure 1:**
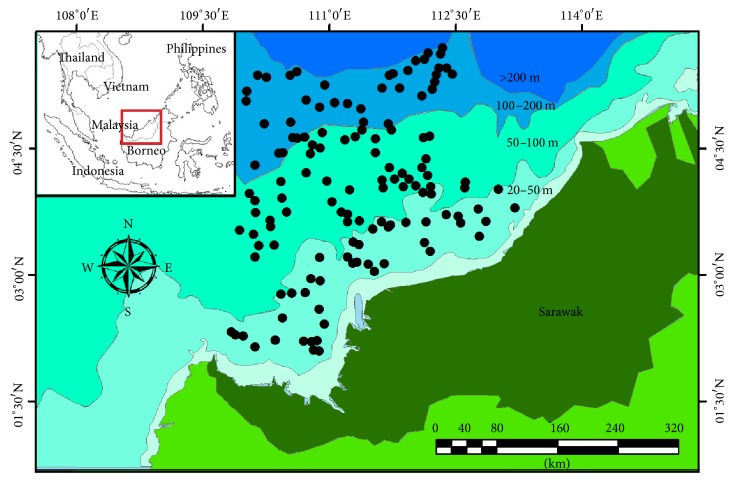
Sampling stations in Sarawak EEZ 2015.

The sea star samples were collected as by-catch during Malaysian EEZ off Sarawak Resource Survey Cruise 2015 (16 Aug–6 Nov 2015). Otter trawl net with a cod-end mesh size of 38 mm had been used throughout the survey with average trawling time of one hour, at preselected stations by DoF M representing different depths (20–200 m) (Figure 1) to ensure fair sampling of the overall EEZ. Collections of samples were done by crews and researchers on the board of MV SEAFDEC 2 vessel, sorted and kept in plastic bags with labels, and stored in −20°C freezer on board. All laboratory works were carried out at the Faculty of Resource Science and Technology, UNIMAS.

Detailed examinations were carried out on 217 specimens, following [1, 11, 14, 15], and then checked against World Register of Marine Species (WoRMS). Morphological features of the samples were observed by naked eye (aboral and oral view) followed by inspection on abactinal (aboral) and actinal (oral) surfaces and terminals (arm tips), whereas detailed observations were done with aid of Olympus SZ 51 stereomicroscope. Characters examined include general body appearance, encrusting ossicles, pedicellariae, madreporite, abactinals, terminals, marginals, intermarginals, actinals, oral frame, ambulacral grooves and interambulacrals, papulae, tube feet, digestive tract, and its development. Other observations include central disc and arms' other characteristics such as pigmentation that present on the oral surface. Photographs were captured using a digital camera.

Feeding behaviour analysis was carried out by examining the stomach contents of* S. childreni* (*n* = 100) with individuals represented each survey station, following methods by [16]. Stomach was dissected using surgical blade. Later, stomach contents were isolated and sorted using forceps. Small hard structures, sediment grains, sclerites or spicules, and aquatic macrophytes were isolated and preserved in 10% formalin (if needed) and labelled. The stomach contents were identified under stereo- and compound-microscope and images were captured. The feeding behaviour analysis was done by calculated prey items from each taxon using following formulae by [17].

(1) $$\begin{array}{c} F_{i}=\left(\;100\;n_{i}\;\right)÷N, \end{array}$$

where, *F*_*i*_ is frequency of occurrence of the *i* food items in* S. children*, *n*_*i*_ is number of stomachs where *i* food items are found, and *N* is total number of stomachs with food in the specimens.

Sea bed characterisations are based on vessel log sheet data provided by the captain of MV SEAFDEC 2 vessel during the field excursion.

## 3. Results

### 3.1. Morphological and Anatomical Examinations

**Figure 2:**
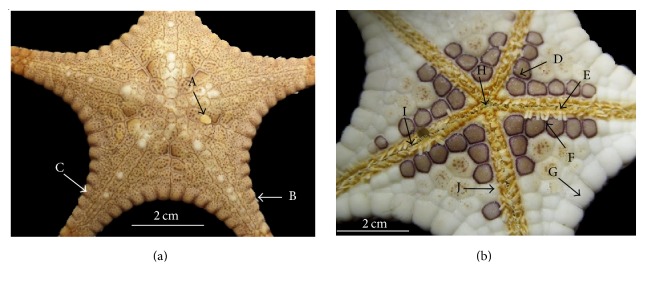
*Stellaster childreni *(Gray, 1840). (a) aboral view, *R* = 66 mm; A: madreporite; B: spine (blunted); C: superomarginal plate; (b) oral view, *r* = 26 mm; D: actinal intermediate areas, with numerous pigmentation lacking suboral spines; E: furrow spines; F: subambulacral spines; G: inferomarginal plate; H: mouth; I: ambulacral groove; J: adambulacral border. *R*/*r* = 66/26 mm. “*R*” is the length of the arm as measured from the center of the animal to the tip of the arm. “*r*” is the radius of the disk and is measured from the center to a notch between the arms.

Morphological examination performed on oral and aboral surfaces of the specimens and recorded in Figure 2.* Stellaster childreni* Gray 1840 displayed typical five arms and comprised well-developed marginal plates which were covered with homogeneous granulation. Blunted white colour spines were distributed of each inferomarginal plate. It was brownish in colour. However, this specimen had a round ornamental with uneven distribution of dark brownish pigmentation throughout the actinal intermediate areas. Massive and complex ornamental and tiny spines appeared at the interradial region. Abactinal plate of each arm contains granules and blunted small spines and pedicellariae. Tube feet were relatively small and embedded inside the adambulacral groove.

In this study, fresh specimens were brownish or muddy colour on aboral surface and white colour on oral surface with brownish pigments.

**Figure 3:**
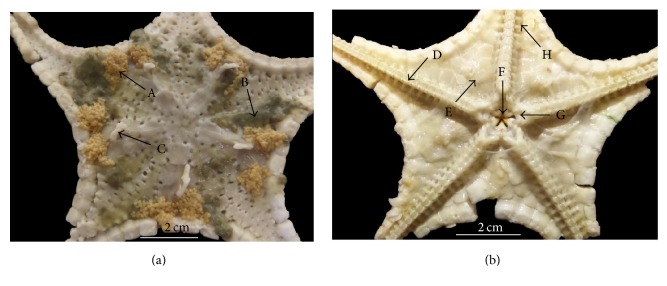
*Stellaster childreni *(Gray, 1840). (a) aboral view, (b) oral surface. A: gonad; B: pyloric ceca; C: spine; D: ambulacral ridge; E: cardiac stomach; F: mouth; G: ring canal; and H: tube feet.

Anatomy examination covered observation on gonad, pyloric ceca, spine, ambulacral ridge, cardiac stomach, mouth, ring canal, and tube feet and photographs were displayed in Figure 3. Stomachs of specimens were filled with sediment (Figure 3), besides the presence of invertebrates such as small bivalves and gastropods. Interradial region comprised pentagonal plates on both aboral and oral surfaces. The stomach has a relatively thin membrane. Tube feet were small and unable to project out, most probably because the specimens had been left in freezing condition for more than one month. Spines were present at the aboral surface. The gonads were yellowish in colour whereas the pyloric caeca appeared brownish in colour.

### 3.2. Diet Analysis

For gut content analysis, about 95.92% of specimens contained food items and sediment in their stomach, whereas 4.08% of specimens had totally empty stomachs. Common food items include juveniles of gastropods and bivalves, barnacle sponge's spicules, fragments of seagrass and seaweed, and benthic Foraminifera (Table 1).

### 3.3. Seabed Types and Depth of Habitats

**Figure 4:**
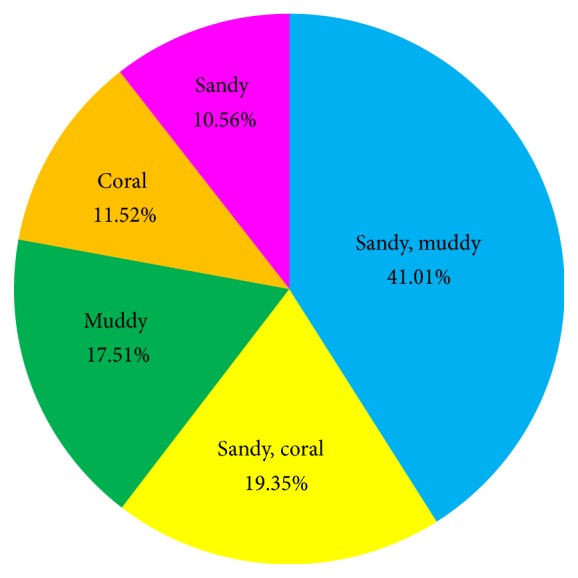
Distribution of* S. childreni *samples according to type of seabed.

About 41.01% of* S. childreni* were found at mixture of sandy and muddy substratum (Figure 4), followed by mixture of sandy and coral (19.35%) substratum, muddy substratum (17.51%), coral substratum (11.52%), and sandy substratum (10.56%). In terms of depth, this study showed that* S. childreni* could live in habitats between 45 m and 185 m.

## 4. Discussion

Morphological and anatomical examinations confirmed that all 217 specimens were* S. childreni *Gray 1841, following [1, 11, 14, 15]. Reference [10] had reported that a novel sea star species name* Stellaster equestris* could be found on seagrass area in the southern Peninsular Malaysia. Detailed examination on photographs and description provided in [10] matched specimens in this study; therefore it is very likely they are the same species. Referring to WoRMS,* S. equestris* Retzius (1820), in fact (1805), is invalid.

The mouth of specimen examined during this study is surrounded by soft and delicate membrane [16–18]. The mouth is directed downwards and opens into a short esophagus. Then the food is passed upward into cardiac stomach. Cardiac stomach is relatively large and oval shaped. Above the cardiac stomach, the pyloric stomach is present while the pyloric caeca are radiated out. Pyloric caeca are the primary storage sites for assimilated food, secrete the digestive enzyme, and absorb the nutrients after digestion. If the size of the prey is large, cardiac stomach is able to evert out through mouth by muscle extending from the wall of the body. The anus is located on the aboral surface, adjacent with the mouth, and looked small and very difficult to observe by naked eyes.

Water vascular system as shown in Figure 3(b) plays important role in locomotion. Seawater is able to enter through madreporite which is a spinous skeletal plate that presents on aboral surface of* S. childreni*. Then water will pass through stone canal, ring canal, and radial canal and finally reach tube feet and ampullae [18]. Tube feet are soft tissues which are embedded within the ambulacral groove [17]. Basically, each tube foot contains sucker at the terminal end tips. Tube feet play important roles in locomotion, feeding, and respiration and as the sensory organ as well. Sucker of tube feet is in contact with the substratum and thus mucus is secreted to help in strengthening the adhesion force and resulted in pushing the body in forwarding movement. In climbing a vertical surface, tube feet pull the body forward. However, on soft muddy and sandy seabed, the sucker of tube feet has less roles because* S. childreni* walks on its own extended tube feet which are like small legs.

Behaviour, morphology, and habitat are among the factors that influence feeding behaviour of sea stars [19]. In this study,* S. childreni* is a predator, which regulates the abundance of species and composition of marine benthic communities.* S. childreni* could also be categorized as mud ingesters [19] and infaunal predators because they swallowed bulk sediment which usually comprised meio- and macrofauna [20]. The ability to swallow bulk sediment is likely because Asteroidea has highly expandable and flexible stomachs [18].

The prey selection will be changed based on the abundance of prey in the niche [21]. About 83.67% of* S. childreni *fed on benthic foraminiferans. Benthic Foraminifera are Protista with the size between 100 and 1000 mm, usually known as bottom dwellers, which inhabit beneath the sea floor [22]. Similarly, [19] had recorded the presence of benthic Foraminifera in the gut of Asteroidea* Mediaster bairdi* and* Ceramaster granularis*. In this study, approximately 61.93% of* S. childreni *fed on molluscs. Molluscs usually burrow and stay inside the sediments and thus matched feeding behaviour of* S. childreni*. Other Asteroidea researchers [21] reported that shelled molluscs (gastropod and bivalve) served as main diet of approximately 90%* Astropecten zebra *and 75% of* A. velitaris*. In this study, detailed examination on the gut content related to molluscs revealed that gastropod juveniles had frequency occurrence of 44.89%, followed by bivalve juveniles (12.24%) and scaphopod juvenile (4.08%).

*Stellaster childreni *lives in the benthic area [23]. During this study, the specimens were found at 45 m to 185 m, comprised various seabed types including sandy, muddy, and coral mixture of sand and mud, and mixture of sand and coral. Reference [8] had reported that 39.0% of their specimens were found in the coral area and followed by sandy bottom and rocks and rubbles. In Southeast coast of India,* S. childreni* were found near muddy sea bed only [4]. Based on WoRMS,* S. childreni* could be found between the ranges of 5 and 190 m depth. Reference [4] had reported that* S. childreni* were caught between depths ranges of 5 to 35 m by bottom trawling activities near the muddy seabed of Southeast coast of India, whereas [8] claimed that most of the sea stars were found between 11 and 15 m.

Since this study only studied involved by-catch invertebrates, there is a possibility of underestimating its actual distribution.

## 5. Conclusions

All specimens examined in this study were* Stellaster childreni. *Diet analysis showed that they fed on various items including molluscs, Foraminifera, sponges, seagrass, and seaweed. In deep sea area, this species inhabits different substrata including muddy, sandy, coral, a mixture of sandy and coral, and mixture of sandy and muddy, from depth 45 m to 185 m. It is very likely that this study underestimates the actual distribution of* S. childreni* in Malaysian EEZ off the Sarawak coast, because individuals were only being collected as by-catch organisms. As sea star is considered as “key stone” species, one may say the EEZ areas surveyed have healthy marine ecosystem; thus further exploration should be carried out to optimize Malaysian deep sea fisheries.

## Figures and Tables

**Table 1:** Frequency of occurrence of prey items in the stomach of *S. childreni*.

Prey item	Phylum		Frequency of occurrence (%)
Kingdom Animalia			
	Phylum Mollusca	Bivalvia	12.24
		Gastropoda	44.89
		Scaphopoda	4.80
	Phylum Porifera	Sponges	4.80
Kingdom Plantae		Seagrass and seaweed	61.22
Kingdom Protista		Foraminifera	83.97
Others		Sediment	95.92
